# Diet and lifestyle in relation to small intestinal cancer risk: findings from the European Prospective Investigation into Cancer and Nutrition (EPIC)

**DOI:** 10.1007/s10552-023-01731-w

**Published:** 2023-06-18

**Authors:** Zeynep Ersoy Guller, Rhea N. Harewood, Elisabete Weiderpass, Inge Huybrechts, Mazda Jenab, José María Huerta, Maria-Jose Sánchez, Paula Jakszyn, Pilar Amiano, Eva Ardanaz, Claudia Agnoli, Rosario Tumino, Domenico Palli, Guri Skeie, Jonas Manjer, Keren Papier, Anne Tjønneland, Anne Kirstine Eriksen, Matthias B. Schulze, Rudolf Kaaks, Verena Katzke, Manuela M. Bergmann, Elio Riboli, Marc J. Gunter, Amanda J. Cross

**Affiliations:** 1 Department of Epidemiology and Biostatistics, School of Public Health, Imperial College London, Norfolk Place, London, W2 1PG UK; 2 Guy’s and St Thomas’ NHS Foundation Trust, Westminster Bridge Road, London, SE1 7EH UK; 3 Nutrition and Metabolism Branch, International Agency for Research on Cancer, Lyon, France; 4 International Agency for Research on Cancer World Health Organization, Lyon, France; 5 CIBER Epidemiología y Salud Pública (CIBERESP), Madrid, Spain; 6 Department of Epidemiology, Murcia Regional Health Council, IMIB-Arrixaca, Murcia, Spain; 7 Escuela Andaluza de Salud Pública (EASP), 18011 Granada, Spain; 8 Instituto de Investigación Biosanitaria ibs. GRANADA, 18012 Granada, Spain; 9 Centro de Investigación Biomédica en Red de Epidemiología y Salud Pública (CIBERESP), 28029 Madrid, Spain; 10 Department of Preventive Medicine and Public Health, University of Granada, 18071 Granada, Spain; 11 Unit of Nutrition and Cancer, Epidemiology Research Program, Catalan Institute of Oncology, Bellvitge Biomedical Research Institute (IDIBELL), Hospitalet de Llobregat, Barcelona, Spain; 12 Blanquerna School of Health Sciences, Ramon Llull University, Barcelona, Spain; 13 Ministry of Health of the Basque Government, Sub Directorate for Public Health and Addictions of Gipuzkoa, San Sebastian, Spain; 14 Biodonostia Health Research Institute, Epidemiology of Chronic and Communicable Diseases Group, San Sebastián, Spain; 15 Spanish Consortium for Research on Epidemiology and Public Health (CIBERESP), Instituto de Salud Carlos III, Madrid, Spain; 16 Navarra Public Health Institute, 31003 Pamplona, Spain; 17 IdiSNA, Navarra Institute for Health Research, 31008 Pamplona, Spain; 18 CIBER Epidemiology and Public Health CIBERESP, 28029 Madrid, Spain; 19 Epidemiology and Prevention Unit, Fondazione IRCCS Istituto Nazionale dei Tumori, Milan, Italy; 20 Hyblean Association for Epidemiological Research AIRE ONLUS, Ragusa, Italy; 21 Cancer Risk Factors and Life-Style Epidemiology Unit, Institute for Cancer Research, Prevention and Clinical Network (ISPRO), Florence, Italy; 22 Department of Community Medicine, UiT The Arctic University of Norway, Tromsø, Norway; 23 Department of Surgery, Lund University, Skåne University Hospital, SE 205 02 Malmö, Sweden; 24 Cancer Epidemiology Unit, Nuffield Department of Population Health, University of Oxford, Oxford, UK; 25 Danish Cancer Society Research Center, Danish Cancer Society, Copenhagen, Denmark; 26 Department of Public Health, University of Copenhagen, Copenhagen, Denmark; 27 Department of Molecular Epidemiology, German Institute of Human Nutrition Potsdam-Rehbruecke, Nuthetal, Germany; 28 Institute of Nutritional Science, University of Potsdam, Nuthetal, Germany; 29 Division of Cancer Epidemiology, German Cancer Research Center (DKFZ), Heidelberg, Germany; 30 German Institute of Human Nutrition Potsdam, Nuthetal, Germany

**Keywords:** Cancer, Small intestine, Adenocarcinoma, Carcinoid, Diet, Lifestyle, Alcohol, Smoking

## Abstract

**Purpose:**

The incidence of small intestinal cancer (SIC) is increasing, however, its aetiology remains unclear due to a lack of data from large-scale prospective cohorts. We examined modifiable risk factors in relation to SIC overall and by histological subtype.

**Methods:**

We analysed 450,107 participants enrolled in the European Prospective Investigation into Cancer and Nutrition cohort. Cox proportional hazards models were used to estimate univariable and multivariable hazard ratios (HRs) and 95% confidence intervals (CIs).

**Results:**

During an average of 14.1 years of follow-up, 160 incident SICs (62 carcinoids, 51 adenocarcinomas) were identified. Whilst univariable models revealed a positive association for current versus never smokers and SIC (HR, 95% CI: 1.77, 1.21–2.60), this association attenuated in multivariable models. In energy-adjusted models, there was an inverse association across vegetable intake tertiles for SIC overall (HR_T3vsT1_, 95% CI: 0.48, 0.32–0.71, p-trend: < 0.001) and for carcinoids (HR_T3vsT1_, 95% CI: 0.44, 0.24–0.82, p-trend: 0.01); however, these attenuated in multivariable models. Total fat was also inversely associated with total SIC and both subtypes but only in the second tertile (SIC univariable HR_T2vsT1_, 95% CI: 0.57, 0.38–0.84; SIC multivariable HR_T2vsT1_, 95% CI: 0.55, 0.37–0.81). Physical activity, intake of alcohol, red or processed meat, dairy products, or fibre were not associated with SIC.

**Conclusion:**

These exploratory analyses found limited evidence for a role of modifiable risk factors in SIC aetiology. However, sample size was limited, particularly for histologic subtypes; therefore, larger studies are needed to delineate these associations and robustly identify risk factors for SIC.

**Supplementary Information:**

The online version contains supplementary material available at 10.1007/s10552-023-01731-w.

## Introduction

The small intestine comprises more than two-thirds of the digestive tract in length and more than 90% of the absorptive surface area [1]. It is situated between the stomach and colon, which are both common sites for cancer to develop [2]; however, the small intestine rarely develops cancer, with incidence ranging between less than 0.5 per 100,000 in some parts of Africa and Asia to 4.1 in specific regions of the United States (US) in the period between 2008 and 2012 [3].

The two main histologic subtypes of small intestinal cancer (SIC) are adenocarcinoma and carcinoid tumours. According to US Surveillance, Epidemiology, and End Results (SEER) registries data, the incidence of SIC increased from 1.16 to 2.52 per 100,000 between 1975 and 2019 [4]; this trend is mainly explained by the 4.4-fold increase in carcinoid tumour incidence, but the underlying aetiological factors remain largely unknown [5]. The increase in incidence has been consistently observed in both sexes and by ethnicity [5]. In concordance, European studies have also reported increased incidence of SIC over the last few decades [6–8]. As a result, it could be hypothesised that the increase in incidence is partly related to changes in modifiable risk factors such as lifestyle and dietary factors [5].

In addition to the anatomic and physiologic similarities, there is further evidence of common causal pathway for adenocarcinoma of the small intestine and colorectum. Firstly, there is a geographical correlation in the incidence of both malignancies, which is attributed to the increases in risk factors associated with the “Westernisation” of diet and lifestyle [9, 10]. Furthermore, the two anatomical sites share the widely acknowledged adenoma-carcinoma sequence of events [11] and there is more than a two-fold increased risk of developing colorectal cancer (CRC) for SIC patients and greater than a three-fold increased risk of developing SIC for CRC patients [12, 13]. These findings suggest that SIC may also share the modifiable risk factors for CRC [1].

While CRC has been extensively studied, the aetiology of SIC remains largely unknown as relatively few epidemiological studies have been conducted due to sample size constraints. Considering the increasing incidence of SIC and the evidence for common causality with CRC, it is important to examine how modifiable risk factors for CRC are associated with SIC. Using the European Prospective Investigation into Cancer and Nutrition (EPIC) cohort, we investigated whether previously identified risk factors for CRC, such as smoking, alcohol, physical activity, and dietary factors such as meat, fat, dairy products, vegetables and fibre are associated with SIC incidence.

## Materials and methods

### Study population

EPIC is an ongoing multicentre prospective cohort study; details of the rationale, design, and data collection methods have been described previously [14, 15]. Between 1992 and 2000, 521,323 participants, mostly between 35 and 70 years of age, were recruited from 23 study centres in ten European countries (Denmark, France, Germany, Greece, Italy, Norway, Spain, Sweden, The Netherlands, and the UK). After exclusion of prevalent cancer cases (*n* = 29,332), participants who did not complete the questionnaires (*n* = 6,259), participants with extreme energy intake (top and bottom 1% based on energy intake to energy requirement ratio or daily energy intake of < 600 kcal or > 6000 kcal) (*n* = 9,577), and participants from Greece (*n* = 26,048) due to data restriction issues, our analytic cohort consisted of 450,107 persons.

At baseline, participants completed detailed questionnaires and anthropometric measurements and blood samples were taken. All participants signed an informed consent agreement, and ethical approval for the EPIC study was provided by the review boards of the International Agency for Research on Cancer (IARC) and local participating centres.

### Assessment of exposures

Diet at baseline was measured with validated questionnaires to measure habitual consumption over the preceding year. Most centres adopted a self-administered quantitative dietary questionnaire of 260 food items while semi-quantitative food frequency questionnaires (FFQs) were used in Denmark, Norway, Naples (Italy), and Umeå (Sweden) and combined dietary methods were used in the UK and Malmö (Sweden). Nutrient intakes were calculated with the use of the EPIC Nutrient DataBase (ENDB), a standardised food-composition table [16, 17]. We examined red meat, processed meat, fibre and dairy as they have either been convincingly or probably associated with CRC [18]. Although the evidence for vegetable and fat intake and CRC risk is limited, we examined these two variables as they have been studied in relation to SIC before [19]. Unfortunately, complete data on whole-grain intake was not available in EPIC, and hence was not included.

A non-dietary questionnaire collected detailed information about alcohol consumption, smoking, physical activity and education at baseline. In most centres, height and body weight were measured at baseline according to standardised procedures; however, in Oxford (UK), France, and Norway, anthropometric values at baseline were self-reported [15]. A study deriving prediction equations from EPIC-Oxford subjects with both standard and self-reported measures showed self-reported measures are valid for identifying relationships in epidemiological studies [20]. A previous analysis in this cohort investigated waist circumference (WC) and body mass index (BMI) in relation to SIC, finding that WC was positively associated with SIC [21].

### Assessment of outcome

Participants were followed for cancer incidence through surveillance of medical records, tumour registry linkage and active follow-up. Follow-up was based on population cancer registries in Denmark, Italy, Netherlands, Norway, Spain, Sweden, and the UK. A combination of other methods, such as health insurance records, cancer and pathology registries and active contact of participants or next of kin were used in France and Germany. Furthermore, the information provided by the participants or their next of kin was verified by physician records. Participants were at risk from their enrollment into the study until diagnosis of SIC, death, loss to follow-up or the end of follow-up (December 2013), whichever occurred first.

The tenth revision of the International Classification of Diseases (ICD-10) and the third edition of the International Classification of Disease for Oncology (ICD-O) were used to code SIC by anatomical location (ICD10: C17.0–17.9) [22, 23]. Analyses by histological subtypes included the two main subtypes of SIC: adenocarcinoma (morphology codes in the EPIC data: 8140/3, 8141/3, 8143/3, 8144/3, 8210/3, 8211/3, 8480/3, and 8481/3) and malignant carcinoid tumours (morphology codes: 8240/3, 8241/3, 8244/3, 8245/3, and 8246/3).

### Statistical analysis

We examined dietary and lifestyle factors in relation to SIC, as well as the subtypes of adenocarcinoma and carcinoid tumours, using Cox proportional hazards models to estimate hazard ratios (HRs) and 95% confidence intervals (95% CIs). Age was used as the underlying timescale in all analyses. All variables had less than 5% missingness except WC, which had 23.5% missing. Those with missing data for a given continuous covariate were automatically removed from that specific model, and missing categories were created for smoking status, education and physical activity.

Univariable and multivariable HRs were reported within predefined categories or tertiles from the whole cohort, using the lowest tertile as the reference category, as well as for continuous data for dietary factors and alcohol consumption. Tests for linear trend within tertiles were performed using the median value of each tertile.

Multivariable models for lifestyle factors were stratified by age (in quintiles), sex and country to control for possible confounding effect of age, differing follow-up methods, questionnaire design, and other variations between countries. For dietary factors, univariable models were adjusted for energy using the nutrient density method while multivariable models were further stratified by age, sex and country [24].

When not the main exposure variable, the following potential confounders were examined based on previous studies on SIC and CRC: BMI (kg/m^2^), WC (cm), height (cm), education (none/primary school, technical/professional, secondary, and university), smoking status (never, former, current), baseline alcohol drinking (g/day), physical activity (inactive, moderately inactive, moderately active, and active, defined by the Cambridge index), intakes of fibre, meat, fish, fruit, vegetables, dairy, calcium and folate. These potential covariates were checked for each multivariable model using the “change-in-estimate” approach in which covariates are selected if their inclusion in a stepwise-manner changes the effect estimate by 10% or more [25]. The confounder selection approach yielded no significant confounding for any of the models.

In sensitivity analyses, we included WC in all multivariable models. In addition, we performed a sensitivity analysis for SIC in which we selected covariates a priori based on evidence from previous studies on SIC and CRC and included all of the following covariates in multivariable models: sex, country, education, smoking status (never, former, current), baseline alcohol drinking (g/day), physical activity (inactive, moderately inactive, moderately active, and active), BMI (kg/m^2^) and energy using the nutrient density method for dietary factors. A lag-analysis was conducted by excluding the 1st year of follow-up to evaluate the potential bias of reverse causality since undiagnosed disease at baseline may have led to changes in diet and lifestyle.

The proportional hazards assumption was tested based on the Schoenfeld residuals. Except for sex, for which the models were stratified by, all the variables fitted the proportionality assumption. Two-sided tests with a significance level of 0.05 were chosen, and all analyses were performed using R version 4.2.2.

## Results

### Basic characteristics

During an average follow-up of 14.1 years, 160 incident SIC cases were identified (66 in men and 94 in women) and they were comprised of 51 adenocarcinomas, 62 carcinoids, 19 sarcomas, 13 lymphomas, and 15 unknown histology. Adenocarcinomas were most commonly found in the duodenum (75.0% of all duodenal cancers were adenocarcinomas and only 2.5% were carcinoids) and jejunum (47.8% of all jejunal cancers were adenocarcinomas, 13.0% were carcinoids). Conversely, carcinoid tumours were mainly located in the ileum (64.6% of ileal cancers were carcinoids and 6.3% were adenocarcinomas).

The average age at study entry was 55.7 years for cases and 51.1 years for non-cases (Table 1). There was a lower percentage of women among cases (58.8%) compared to non-cases (70.8%). The distributions of baseline characteristics are given in Table 1 by sex.

**Table 1:** Baseline characteristics of small intestinal cancer cases and non-cases in EPIC (*n* = 450,107)

Variable	Small intestinal cancer cases		Non-cases	
	*(n* = *160)*		*(n* = *449,947)*	
	Men *(n* = *66)*	Women *(n* = *94)*	Men *(n* = *131,356)*	Women *(n* = *318,591)*
Age at recruitment, years^a^	58.4 (7.56)	53.9 (8.27)	52.2 (9.89)	50.7 (9.66)
Education, *n* (%)^b^				
None/primary school	26 (39.4)	32 (34.4)	41,938 (32.2)	84,618 (27.0)
Technical/professional school	15 (22.7)	22 (23.7)	32,641 (25.1)	71,101 (22.7)
Secondary school	8 (12.1)	16 (17.2)	17,441 (13.4)	76,445 (24.4)
University degree	17 (25.8)	22 (23.7)	35,506 (27.3)	73,386 (23.4)
Smoking status, *n* (%)^b^				
Never	18 (27.3)	45 (47.9)	44,191 (33.6)	175,038 (54.9)
Former	27 (40.9)	22 (23.4)	48,252 (36.7)	74,378 (23.3)
Current	20 (30.3)	25 (26.6)	37,531 (28.6)	62,137 (19.5)
Body mass index (kg/m^2^)^a^	27.1 (3.74)	24.5 (4.45)	26.4 (3.62)	24.8 (4.32)
Waist circumference, cm^a^	97.9 (10.8)	80.0 (11.1)	94.3 (10.1)	79.6 (11.2)
Height, cm^a^	176.8 (7.56)	163.4 (5.95)	175.1 (7.24)	162.6 (6.58)
Cambridge physical activity index, *n* (%)^b^				
Inactive	13 (19.7)	26 (27.7)	23,061 (17.6)	64,930 (20.4)
Moderately inactive	23 (34.8)	30 (31.9)	40,622 (30.9)	109,265 (34.3)
Moderately active	16 (24.2)	20 (21.3)	31,662 (24.1)	88,500 (27.8)
Active	13 (19.7)	18 (19.1)	32,939 (25.1)	50,145 (15.7)
Alcohol				
Alcohol (g/day)^a^	21.6 (23.3)	8.5 (12.6)	20.5 (22.9)	8.1 (11.7)
Never drinkers, *n* (%)	1 (1.5)	3 (3.2)	1248 (1.0)	22,053 (6.9)
Dietary variables				
Fibre (g/1000 kcal)^a^	9.6 (2.5)	11.8 (3.7)	10.3 (3.0)	11.7 (3.3)
Fat (g/1000 kcal)^a^	38.3 (7.0)	38.1 (6.8)	38.0 (6.5)	38.6 (6.5)
Red meat (g/1000 kcal)^a^	25.1 (17.0)	20.2 (14.9)	22.6 (16.7)	19.5 (15.7)
Processed meat (g/1000 kcal)^a^	18.7 (13.5)	13.4 (11.5)	18.4 (14.5)	14.9 (12.4)
Dairy (g/1000 kcal)^a^	158.7 (102.0)	182.6 (103.8)	146.6 (109.8)	172.6 (111.9)
Vegetables (g/1000 kcal)^a^	60.0 (40.9)	101.2 (88.0)	73.1 (53.2)	110.8 (70.3)
Calcium (mg/day)^a^	1072 (388)	1009 (355)	1041 (430)	977 (402)
Folate (microgram/day)^a^	310 (105)	298 (132)	315 (116)	303 (122)
Energy (kcal/day)^a^	2506 (658)	2040 (554)	2417 (662)	1936 (541)

^a^Reported as mean and standard deviation

^b^Numbers do not add up to 100% due to missing data

### Lifestyle risk factors

In the univariable model, current smokers had an elevated risk of SIC compared to never-smokers (HR, 95% CI: 1.77, 1.21–2.60) but the association attenuated once it was stratified by age, sex and country (HR, 95% CI: 1.29, 0.87–1.92) (Table 2). HRs were elevated but not statistically significant for current smoking and risk of both histological subtypes of SIC. There were no associations between alcohol consumption or levels of physical activity in relation to the risk of SIC, adenocarcinoma or carcinoid tumours (Table 2).

**Table 2:** HRs and 95% CIs for small intestinal cancer risk in relation to lifestyle factors

	Small intestinal cancer (*n* = 160)		Adenocarcinoma (*n* = 51)		Carcinoid tumour (*n* = 62)	
	Univariable model HR (95% CI)	Multivariable model^a^ HR (95% CI)	Univariable model HR (95% CI)	Multivariable model^a^ HR (95% CI)	Univariable model HR (95% CI)	Multivariable model^a^ HR (95% CI)
Alcohol (g/day)						
T1 (0.35)^b^	Ref	Ref	Ref	Ref	Ref	Ref
T2 (5.49)^b^	0.96 (0.65–1.43)	0.84 (0.56–1.27)	0.63 (0.31–1.29)	0.54 (0.26–1.12)	1.00 (0.54–1.84)	0.86 (0.46–1.61)
T3 (22.86)^b^	1.23 (0.85–1.79)	1.02 (0.68–1.55)	0.97 (0.51–1.82)	0.79 (0.39–1.59)	0.98 (0.54–1.81)	0.91 (0.46–1.80)
p trend	0.19	0.66	0.78	0.86	0.96	0.89
Continuous (5 g/day)	1.04 (1.00–1.08)	1.03 (0.98–1.07)	1.03 (0.96–1.11)	1.02 (0.94–1.11)	0.99 (0.91–1.07)	0.98 (0.89–1.08)
Smoking						
Never	Ref	Ref	Ref	Ref	Ref	Ref
Former	1.32 (0.91–1.92)	1.03 (0.70–1.52)	1.19 (0.61–2.31)	0.98 (0.50–1.95)	1.28 (0.69–2.35)	1.01 (0.54–1.89)
Current	1.77 (1.21–2.60)	1.29 (0.87–1.92)	1.82 (0.94–3.55)	1.47 (0.74–2.94)	1.76 (0.95–3.29)	1.30 (0.68–2.46)
Physical activity						
Inactive	Ref	Ref	Ref	Ref	Ref	Ref
Moderately inactive	0.94 (0.62–1.42)	0.81 (0.53–1.24)	1.12 (0.52–2.41)	0.99 (0.45–2.16)	0.91 (0.47–1.79)	0.77 (0.39–1.53)
Moderately active	0.91 (0.58–1.45)	0.70 (0.44–1.14)	1.08 (0.46–2.53)	0.92 (0.38–2.22)	1.20 (0.60–2.41)	0.86 (0.41–1.79)
Active	1.09 (0.67–1.75)	0.78 (0.47–1.29)	1.61 (0.70–3.72)	1.15 (0.47–2.78)	0.73 (0.31–1.73)	0.57 (0.23–1.40)

*HR* hazard ratio, 95% CI 95% confidence interval, *Ref* reference category

^a^Stratified by age, sex and country

^b^Median value of tertile

### Dietary risk factors

In univariable models, there was an inverse association in the highest tertile of vegetable intake for SIC overall (HR, 95% CI: 0.48, 0.32–0.71, p-trend: < 0.001) and for continuous data (HR, 95% CI: 0.67, 0.50–0.90, per 100 g/1000 kcal increase) that remained statistically significant in the multivariable model for the medium versus lowest tertile only (HR, 95% CI: 0.64, 0.44–0.94, p-trend: 0.23) (Table 3). A similar inverse association was observed in the univariable model for carcinoid tumours (HR_T3vsT1_, 95% CI: 0.44, 0.24–0.82, p-trend: 0.01; HR_continous_, 95% CI: 0.49, 0.29–0.83) that remained significant in the multivariable model for the medium versus lowest tertile only (HR, 95% CI: 0.46, 0.23–0.88, p-trend: 0.37; Table 3).

**Table 3:** HRs and 95% CIs for small intestinal cancer risk in relation to meat, dairy, and vegetable intake

	Small intestinal cancer (*n* = 160)		Adenocarcinoma (*n* = 51)		Carcinoid tumour (*n* = 62)	
	Univariable model HR (95% CI)	Multivariable model^a^ HR (95% CI)	Univariable model HR (95% CI)	Multivariable model^a^ HR (95% CI)	Univariable model HR (95% CI)	Multivariable modela HR (95% CI)
Red meat (g/1000 kcal)						
T1 (≤ 11.1)	Ref	Ref	Ref	Ref	Ref	Ref
T2 (> 11.1, ≤ 24.8)	0.97 (0.66–1.44)	0.92 (0.62–1.37)	0.77 (0.38–1.54)	0.75 (0.37–1.53)	0.76 (0.41–1.41)	0.76 (0.41–1.44)
T3 (> 24.8, ≤ 252)	0.97 (0.66–1.43)	0.99 (0.63–1.55)	0.90 (0.46–1.75)	0.84 (0.39–1.84)	0.79 (0.43–1.44)	1.09 (0.54–2.19)
p trend	0.91	0.99	0.85	0.73	0.51	0.77
Continuous (10 g/1000 kcal)	1.03 (0.93–1.13)	1.04 (0.93–1.17)	1.02 (0.86–1.21)	1.01 (0.83–1.23)	0.97 (0.83–1.14)	1.07 (0.89–1.29)
Processed meat (g/1000 kcal)						
T1 (≤ 8.68)	Ref	Ref	Ref	Ref	Ref	Ref
T2 (> 8.68, ≤ 18.4)	0.96 (0.66–1.40)	0.82 (0.55–1.21)	0.65 (0.33–1.29)	0.57 (0.28–1.15)	0.74 (0.39–1.39)	0.58 (0.30–1.11)
T3 (> 18.4, ≤ 196)	1.06 (0.72–1.55)	0.71 (0.46–1.09)	0.86 (0.45–1.65)	0.65 (0.31–1.35)	1.16 (0.64–2.08)	0.63 (0.33–1.23)
p trend	0.74	0.13	0.72	0.30	0.51	0.26
Continuous (10 g/1000 kcal)	1.02 (0.90–1.16)	0.89 (0.77–1.03)	0.86 (0.66–1.11)	0.75 (0.55–1.01)	1.14 (0.96–1.36)	0.98 (0.78–1.21)
Dairy (g/1000 kcal)						
T1 (≤ 103)	Ref	Ref	Ref	Ref	Ref	Ref
T2 (> 103, ≤ 193)	1.02 (0.68–1.53)	1.08 (0.72–1.63)	0.48 (0.22–1.03)	0.47 (0.22–1.04)	1.18 (0.61–2.27)	1.25 (0.64–2.44)
T3 (> 193, ≤ 1.58e + 03)	1.25 (0.85–1.83)	1.33 (0.88–2.00)	0.95 (0.51–1.79)	0.82 (0.42–1.60)	1.44 (0.76–2.72)	1.67 (0.86–3.25)
p trend	0.23	0.16	0.85	0.83	0.25	0.12
Continuous (100 g/1000 kcal)	1.03 (0.89–1.18)	1.04 (0.90–1.20)	1.09 (0.87–1.38)	1.04 (0.81–1.33)	0.98 (0.78–1.23)	1.02 (0.81–1.30)
Vegetables (g/1000 kcal)						
T1 (≤ 62.9)	Ref	Ref	Ref	Ref	Ref	Ref
T2 (> 62.9, ≤ 111)	0.55 (0.38–0.80)	0.64 (0.44–0.94)	0.60 (0.30–1.20)	0.73 (0.35–1.48)	0.37 (0.20–0.70)	0.46 (0.23–0.88)
T3 (> 111, ≤ 1.21e + 03)	0.48 (0.32–0.71)	0.76 (0.48–1.20)	0.79 (0.41–1.53)	1.43 (0.67–3.05)	0.44 (0.24–0.82)	0.73 (0.36–1.49)
p trend	< 0.001	0.23	0.60	0.32	0.01	0.37
Continuous (100 g/1000 kcal)	0.67 (0.50–0.90)	0.98 (0.71–1.35)	0.83 (0.52–1.32)	1.17 (0.72–1.92)	0.49 (0.29–0.83)	0.72 (0.40–1.30)

*HR* hazard ratio, *95% CI* 95% confidence interval, *Ref* reference category

^a^ Adjusted for energy, stratified by age, sex and country

Total fat was inversely associated with SIC only in the middle versus lowest tertile (univariable HR_T2vsT1_, 95% CI: 0.57, 0.38–0.84; multivariable HR_T2vsT1_, 95% CI: 0.55, 0.37–0.81; Table 4) and was consistent across both histologic subtypes (adenocarcinoma multivariable HR_T2vsT1_, 95% CI: 0.44, 0.22–0.91; carcinoid multivariable HR_T2vsT1_, 95% CI: 0.44, 0.22–0.87; Table 4). There were no clear associations by fat subtype. Similar to total fat, monounsaturated fat was inversely associated with SIC only in the middle versus lowest tertile in the multivariable model (HR_T2vsT1_, 95% CI: 0.63, 0.42–0.93; Table 4). Although there was an inverse association in the univariable model for polyunsaturated fat and SIC in the continous data (HR, 95% CI: 0.40, 0.18–0.90, per 10 g/1000 kcal increase), this attenuated in the multivariable model. We did not observe any associations for red meat, processed meat, dairy, fibre or saturated fat intake (Tables 3, 4).

**Table 4:** HRs and 95% CIs for small intestinal cancer risk in relation to fibre, fat and its sub-groups

	Small intestinal cancer (*n* = 160)		Adenocarcinoma (*n* = 51)		Carcinoid tumour (*n* = 62)	
	Univariable model HR (95% CI)	Multivariable model^a^ HR (95% CI)	Univariable model HR (95% CI)	Multivariable model^a^ HR (95% CI)	Univariable model HR (95% CI)	Multivariable model^a^ HR (95% CI)
Fibre (g/1000 kcal)						
T1 (≤ 9.7)	Ref	Ref	Ref	Ref	Ref	Ref
T2 (> 9.7, ≤ 12.2)	1.03 (0.72–1.48)	1.15 (0.79–1.68)	0.69 (0.35–1.37)	0.78 (0.39–1.57)	0.96 (0.53–1.74)	1.09 (0.59–2.03)
T3 (> 12.2, ≤ 47.8)	0.78 (0.51–1.17)	0.93 (0.60–1.44)	0.78 (0.40–1.54)	0.94 (0.46–1.95)	0.84 (0.44–1.60)	1.06 (0.53–2.11)
p trend	0.22	0.74	0.49	0.88	0.59	0.87
Continuous (10 g/1000 kcal)	0.77 (0.46–1.31)	1.05 (0.61–1.82)	0.71 (0.28–1.78)	0.94 (0.36–2.46)	0.74 (0.32–1.72)	1.08 (0.44–2.63)
Total fat (g/1000 kcal)						
T1 (≤ 35.7)	Ref	Ref	Ref	Ref	Ref	Ref
T2 (> 35.7, ≤ 41.1)	0.57 (0.38–0.84)	0.55 (0.37–0.81)	0.45 (0.22–0.93)	0.44 (0.22–0.91)	0.46 (0.23–0.91)	0.44 (0.22–0.87)
T3 (> 41.1, ≤ 80.9)	0.76 (0.53–1.09)	0.72 (0.50–1.05)	0.65 (0.34–1.23)	0.63 (0.32–1.22)	0.90 (0.51–1.58)	0.84 (0.47–1.51)
p trend	0.12	0.07	0.15	0.14	0.69	0.54
Continuous (10 g/1000 kcal)	0.91 (0.72–1.16)	0.88 (0.68–1.13)	0.94 (0.62–1.44)	0.93 (0.60–1.45)	0.97 (0.66–1.43)	0.91 (0.61–1.37)
Saturated fat (g/1000 kcal)						
T1 (≤ 13.3)	Ref	Ref	Ref	Ref	Ref	Ref
T2 (> 13.3, ≤ 16.3)	1.00 (0.67–1.48)	0.87 (0.58–1.31)	0.72 (0.36–1.43)	0.56 (0.28–1.14)	1.16 (0.61–2.21)	0.99 (0.51–1.93)
T3 (> 16.3, ≤ 43)	1.14 (0.78–1.66)	0.95 (0.63–1.44)	0.87 (0.45–1.66)	0.61 (0.30–1.23)	1.37 (0.74–2.54)	1.12 (0.57–2.21)
p trend	0.50	0.85	0.67	0.19	0.32	0.72
Continuous (10 g/1000 kcal)	1.17 (0.77–1.76)	0.93 (0.58–1.49)	1.37 (0.67–2.84)	1.00 (0.44–2.28)	1.28 (0.66–2.49)	0.96 (0.45–2.05)
Monounsaturated fat (g/1000 kcal)						
T1 (≤ 12.1)	Ref	Ref	Ref	Ref	Ref	Ref
T2 (> 12.1, ≤ 14.6)	0.68 (0.46–1.01)	0.63 (0.42–0.93)	0.58 (0.30–1.14)	0.56 (0.28–1.10)	0.83 (0.44–1.56)	0.74 (0.39–1.41)
T3 (> 14.6, ≤ 44.2)	0.78 (0.54–1.14)	0.72 (0.47–1.10)	0.59 (0.30–1.16)	0.60 (0.28–1.29)	1.04 (0.57–1.90)	0.99 (0.51–1.94)
p trend	0.24	0.14	0.13	0.18	0.84	0.97
Continuous (10 g/1000 kcal)	0.84 (0.52–1.35)	0.79 (0.43–1.43)	0.69 (0.28–1.66)	0.84 (0.28–2.50)	1.01 (0.48–2.13)	1.03 (0.40–2.69)
Polyunsaturated fat (g/1000 kcal)						
T1 (≤ 5.3)	Ref	Ref	Ref	Ref	Ref	Ref
T2 (> 5.3, ≤ 6.98)	0.98 (0.69–1.41)	0.91 (0.62–1.32)	0.76 (0.40–1.46)	0.71 (0.36–1.40)	0.95 (0.54–1.67)	0.83 (0.46–1.49)
T3 (> 6.98, ≤ 38)	0.70 (0.47–1.04)	0.72 (0.47–1.11)	0.70 (0.35–1.38)	0.72 (0.35–1.50)	0.61 (0.32–1.18)	0.61 (0.31–1.23)
p trend	0.07	0.13	0.31	0.41	0.14	0.17
Continuous (10 g/1000 kcal)	0.40 (0.18–0.90)	0.44 (0.17–1.10)	0.51 (0.13–2.08)	0.60 (0.12–2.96)	0.35 (0.09–1.33)	0.35 (0.08–1.67)

*HR* hazard ratio, *95% CI* 95% confidence interval, *Ref* reference category

^a^Adjusted for energy, stratified by age, sex and country

The exclusion of the first year of follow-up eliminated 10 SIC cases (3 adenocarcinomas and 2 carcinoids) and the results were not materially different from the main findings (data not shown). In sensitivity analyses, further adjustment for WC did not change the results substantially (Supplementary Tables 1–3); however, it revealed an inverse association for processed meat and SIC adenocarcinoma in both categorical (HR_T3vsT1_, 95% CI: 0.41, 0.17–0.95, p-trend: 0.04) and in continous data for multivariable models (HR, 95% CI: 0.64, 0.45–0.91, per 10 g/1000 kcal increase; Supplementary Table 2). Multivariable models containing all a priori selected covariates did not materially change our results for SIC (data not shown).

## Discussion

In this large cohort of European adults, we found a suggestive positive association for smoking and suggestive inverse associations for vegetables and total fat intake with SIC. However, our sample size was limited, particularly in analyses stratified by histologic subtype. In agreement with existing evidence from other cancer databases [26], the most common histological subtype in our study was carcinoid tumours (39%) followed by adenocarcinomas (32%), with adenocarcinomas occurring most frequently in the duodenum and carcinoid tumours in the ileum.

Although the literature is still limited for SIC, there is now strong evidence for the positive association between smoking and CRC [27]. In our univariable model, current smokers had an elevated risk of SIC but the association attenuated upon stratification by age, sex and country. Our findings support the meta-analysis (28) of four case–control studies [29–32] and one prospective cohort study [33] investigating small intestinal adenocarcinoma risk, which yielded a non-significant pooled risk ratio (RR) of 1.24 (95% CI: 0.71–2.17) for those in the highest versus lowest category of smoking. A previous European case–control study suggested that ever being a smoker was positively associated with carcinoid tumours in the small intestine [34]; however, a case–control [35] and a cohort study [33] did not replicate these results.

According to the IARC Monographs, there is convincing evidence to conclude that alcohol consumption is causally related to CRC risk, but the evidence is not sufficient for SIC [27]. Our study found no associations for alcohol and SIC, which is in agreement with a meta-analysis of small intestinal adenocarcinoma [28] of four case–control studies [29–32] and one cohort study [33]. Evidence is sparse for the role of alcohol consumption for carcinoid tumours, with one case–control study [29] that suggested a positive association, while two case–control studies [34, 35] and a cohort study [33] reported null results. 

There is consistent evidence of a protective association between physical activity and CRC [18, 36, 37]; however, we observed no associations between physical activity and SIC overall or by subtype. The only other cohort study investigating physical activity in relation to SIC studied adenocarcinoma specifically and found no association [33]. 

While obesity is an established risk factor for CRC, a meta-analysis of cohort studies showed that the association is more sensitive to anthropometric indexes of abdominal obesity than to overall obesity [38]. Similarly, the results from the only prospective cohort study that investigated the role of obesity in SIC using EPIC data suggested that abdominal obesity (measured by WC), rather than overall adiposity (measured by BMI), was positively associated with SIC, specifically for adenocarcinomas [21]. However, the interpretability of results was limited due to an even smaller number of cases than observed in this current study.

While there is little evidence in the literature for an association between red meat or processed meat intake and SIC, there is a considerable amount of data suggesting a positive association for cancers of the colorectum [39], oesophagus and stomach [40, 41]. The current analysis found no significant association for red or processed meat and SIC, except for an inverse association for processed meat and adenocarcinoma that was only observed in sensitivity analyses upon further adjustment for WC. The only other cohort study that explored these dietary factors reported no associations between red or processed meats and SIC [19]. There are case–control studies [31, 32, 42] that have reported significantly increased risks of SIC with consumption of red and processed meat; however, these are subject to various biases and two were small in size [31, 32].

Evidence for the association between vegetable intake and SIC is limited to a case–control study [31], which reported a reduced risk of small intestinal adenocarcinoma for individuals with high intake of vegetables. This is the first prospective cohort study examining the association between vegetable intake and SIC risk. In our univariable models for vegetable intake, the risk of SIC and carcinoid tumours was significantly decreased across the tertiles and in continuous data but these associations attenuated in multivariable models.

Although some studies [43, 44] support a positive association between dietary fat and CRC risk, the evidence for fat and its subtypes in relation to SIC is limited to one other cohort study [19]. This previous study in the US reported a positive association between saturated fat consumption and carcinoid tumours, a suggestive elevation in risk for adenocarcinoma with polyunsaturated fat intake, but no association for monounsaturated fat. In contrast, our study yielded an inverse association for total fat in relation to SIC, adenocarcinoma and carcinoid tumour but only in the middle compared to the lowest tertile of intake. Although the inverse association was also observed in the middle category of monounsaturated fat intake and SIC overall in multivariable models, the findings by subtype in our data were not statistically significant in either group. An important potential difference between the data from the US and Europe is the likely different sources of fat. In Europe, particularly Southern Europe, significant sources of monounsaturated fat intake would include olive oil [45], whereas in the US it would include French fries, potato chips, whole milk and ground beef in adults [46].

Studies investigating the association between dietary fibre intake and CRC risk have yielded inconsistent results [43, 47, 48], but inverse associations for whole grains specifically in relation to CRC are more robust [49]. We observed no associations for fibre and SIC, which is in agreement with a previous prospective cohort study [50]. Unfortunately, we were unable to estimate intake of whole grains in EPIC.

The strengths of the present study include the large size and long follow-up of the cohort, which has allowed us to study lifestyle and dietary risk factors by histological subtypes of SIC. The collection of exposure information at baseline and comprehensive follow-up through tumour registry linkage and/or active follow-up provided valuable information about temporality, minimised recall bias and reduced selection bias. However, this study lacked time-varying information on exposures and covariates as these were only measured at baseline. Other limitations included potential measurement error due to use of dietary data from self-reported questionnaires and the relatively small number of incident cases, which restricted the power to detect associations and the interpretability of results, especially by histological subtypes. It should be noted that the findings of this study are exploratory, and they should be interpreted cautiously. Nevertheless, this study is valuable as it is one of the few prospective cohort studies investigating diet and lifestyle factors in relation to SIC and its histological subtypes.

In summary, the epidemiological evidence for dietary and lifestyle risk factors for SIC is limited to mostly case–control studies and only a handful of prospective cohort studies. Therefore, the exploration of these risk factors in EPIC provides valuable insight with its prospective design and large sample size. This study revealed suggestive inverse associations for vegetable intake with SIC and carcinoid tumour risk as well as suggestive inverse associations for total fat with SIC overall and by histological subtypes. Additional research is needed to investigate the associations with a larger number of cases, which could be achieved by pooling existing studies with relevant data in order to suggest preventive strategies for SIC.

## Supplementary Information

Below is the link to the electronic supplementary material.

Supplementary file1 (XLSX 12 KB)

Supplementary file2 (XLSX 13 KB)

Supplementary file3 (XLSX 13 KB)

## Data Availability

The EPIC study data can be accessed via an application to the EPIC Steering Committee (https://epic.iarc.fr/access/index.php). Further information is available from the corresponding author upon request.

## Code availability

Computer code used to generate results is available to editors and reviewers from the corresponding author upon request.
